# OpenVar: functional annotation of variants in non-canonical open reading frames

**DOI:** 10.1186/s13578-022-00871-x

**Published:** 2022-08-14

**Authors:** Marie A. Brunet, Sébastien Leblanc, Xavier Roucou

**Affiliations:** 1 Department of Pediatrics, Medical Genetics Service, Université de Sherbrooke, 3201 Jean Mignault, Sherbrooke, QC J1E 4K8 Canada; 2 Centre de Recherche du Centre Hospitalier Universitaire de Sherbrooke (CRCHUS), Sherbrooke, QC Canada; 3 Department of Biochemistry and Functional Genomics, Université de Sherbrooke, 3201 Jean Mignault, Sherbrooke, QC J1E 4K8 Canada; 4 Quebec Network for Research on Protein Function, Structure, and Engineering, PROTEO, Quebec, Canada

**Keywords:** Small ORF, Alternative ORF, Variant annotation, NGS, Genome, SNP, OpenProt

## Abstract

**Background:**

Recent technological advances have revealed thousands of functional open reading frames (ORF) that have eluded reference genome annotations. These overlooked ORFs are found throughout the genome, in any reading frame of transcripts, mature or non-coding, and can overlap annotated ORFs in a different reading frame. The exploration of these novel ORFs in genomic datasets and of their role in genetic traits is hindered by a lack of software.

**Results:**

Here, we present OpenVar, a genomic variant annotator that mends that gap and fosters meaningful discoveries. To illustrate the potential of OpenVar, we analysed all variants within SynMicDB, a database of cancer-associated synonymous mutations. By including non-canonical ORFs in the analysis, OpenVar yields a 33.6-fold, 13.8-fold and 8.3-fold increase in high impact variants over Annovar, SnpEff and VEP respectively. We highlighted an overlapping non-canonical ORF in the *HEY2* gene where variants significantly clustered.

**Conclusions:**

OpenVar integrates non-canonical ORFs in the analysis of genomic variants, unveiling new research avenues to better understand the genotype–phenotype relationships.

## Background

Technological advances have recently enabled both routine whole-genome sequencing and a deeper understanding of the coding potential of genomes. On the former, with the introduction of Next-Generation Sequencing techniques, reading one’s genome has never been so reliable and affordable, still a fourth of Mendelian phenotypes remain without a known molecular basis (www.omim.org/statistics). On the latter, the emergence of ribosome profiling (Ribo-seq) has unveiled the unsuspected depth of the coding potential of eukaryotic genomes [1]. As Ribo-seq detects mRNA translation events, it revealed signatures outside of annotated open reading frames (ORFs) defined by reference gene annotations [2]. Yet, these annotations are used worldwide in fundamental and clinical research, providing the necessary dictionary to read a genome. Now aware that thousands of functional alternative ORFs have eluded annotation [3–6], we need to assess their role in diseases [7–9].

Serendipitous discoveries have highlighted likely pathological variants in currently unannotated ORFs [7, 8, 10]. However, most studies focused on upstream ORFs [8], partly because no tool is currently available to evaluate variants falling in dual-coding regions where two ORFs overlap. This lack of software drastically impedes the discoveries and interpretation of disease-linked variants in these regions [10].

Therefore, we developed OpenVar (https://openprot.org/openvar/), an open source tool to annotate and explore the impact of genomic variants using deeper genome annotations, such as OpenProt [3]. Genomic variants encompass single nucleotide polymorphisms (SNP), insertions and deletions (INDELs), and multiple nucleotide polymorphisms (MNP). OpenVar annotates such genomic variants both based on their locus (e.g. upstream/downstream, intronic, etc.) and their effect on all coding sequences annotated in OpenProt (e.g. amino acid substitution, stop gain/loss, etc.). OpenVar is available as a python package and an easy-to-use web-based platform, facilitating its implementation independently of one’s computing knowledge or resources.

## Results

**Fig. 1:**
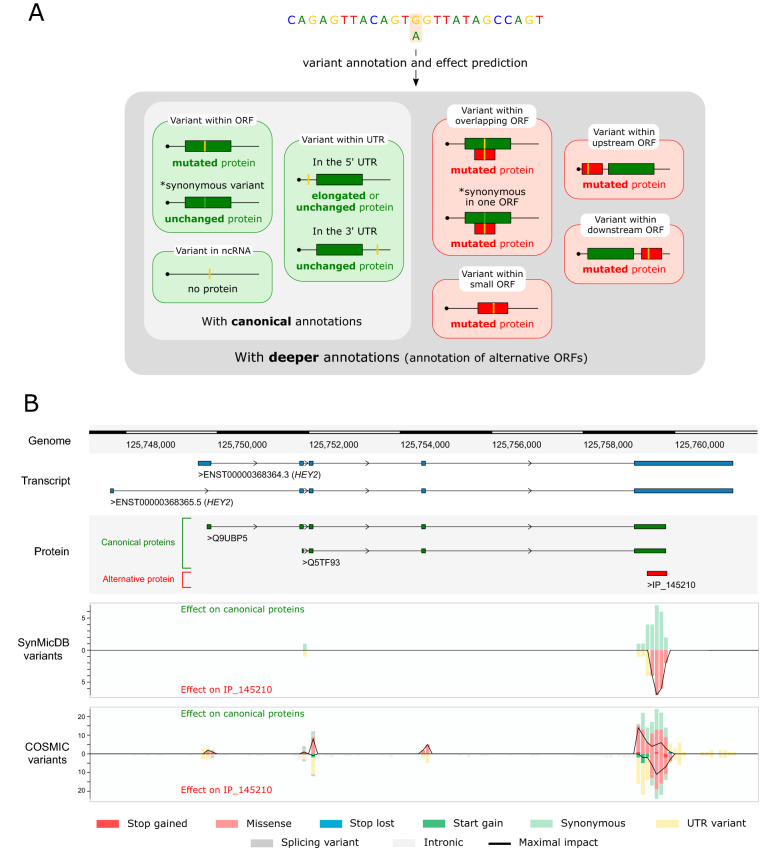
OpenVar to annotate and explore the impact of genomic variants using deeper genome annotations. **A** OpenVar uses a deep genome annotation, OpenProt, to annotate genomic variants (yellow). It will annotate the effect on canonical ORFs (green) listed in reference annotations, and on alternative ORFs (red) listed in OpenProt. For example, a given variant may be a 3′UTR variant for a canonical ORF, but a missense variant for a downstream ORF. **B** Visualization of the genomic variants in the *HEY2* gene from the SynMicDB and COSMIC datasets. The genomic position is indicated at the top (genome), with the *HEY2* transcripts in blue (Ensembl: ENST00000368364.3 and ENST00000368365.5; respectively NCBI RefSeq: NM_012259.2 and XM_017010629.2), the canonical ORFs in green (UniProt: Q9UBP5 and Q5TF93; respectively Ensembl: ENSP00000357348.3 and ENSP00000357349.1; or NCBI RefSeq: NP_036391.1 and XP_016866118.1) and an alternative ORF in red (OpenProt: IP_145210). The variants tracks are below (SynMicDB and COSMIC variants), each grouping the variants per category of effect on the canonical proteins (up side) and the alternative protein (flipped). The colour code for the effect of the variants is at the bottom of the figure. The black line on the variants tracks indicates the number of variants with the maximal impact between the canonical and the alternative ORFs

OpenVar adopts the widely used SnpEff code [11] and the deep genome annotation OpenProt [3] to annotate a variant calling file (VCF). To handle multiple ORFs on a single transcript, OpenVar combines the transcript and protein accession as a unique identifier. This strategy allows the visualization, for a single variant, of all consequences across every transcript and every ORF it affects (Fig. 1A). Alongside the common annotated VCF format, OpenVar outputs a table listing all effects for each variant submitted in the initial VCF. This table is named “annOnePerLine.tsv” as it contains one line for each effect of each variant. It lists the chromosome and position of the variant, the reference and alternative alleles, the description of the effect of the variant, the impact category, the transcript and protein accession, alongside the gene name. To ease interpretation of a much more complex annotation, OpenVar also outputs a table, named “max_impact.tsv”, listing the most affected canonical and non-canonical ORFs for each variant. The prioritization of effects is based on the predicted impact of the variant on the ORF of interest. Impacts are categorized as “modifier”, “low”, “moderate” and “high”. This classification is identical to that used in the SnpEff algorithm [11]. For a non-annotated ORF to be reported in the maximal impact table, a given variant must have a greater or identical impact on it than on a canonical ORF. OpenVar always reports the effect on canonical ORFs in each of its outputs. Thus, the effect of a given variant on a canonical ORF is always visible, but users also see the effect on any alternative ORF (Fig. 1A).

By offering deep annotation of genomic variants, OpenVar can help make meaningful discoveries. Here, we have used it to explore a specific class of variants in cancers. We took advantage of the recently published SynMic database [12]. The database lists variants leading to synonymous mutations in canonical proteins across 18,028 samples from 88 different tumor entities. Interestingly, synonymous mutations are the second most frequent in cancer samples behind missense mutations [12]. The mechanisms behind their pathological impact remain unknown for most synonymous mutations. Current theories revolve around splicing junctions and transcript structure and/or stability [10, 13]. However, some of these variants may also have a greater impact on an overlapping ORF as recently demonstrated [7].

To illustrate the potential of OpenVar, we analysed all the SynMicDB variants with it and we highlighted an overlapping alternative ORF in the *HEY2* gene. 26 mutations where reported within *HEY2*, all falling within the union of the canonical ORF (Q9UBP5) and an alternative ORF (IP_145210) detected with a unique peptide by mass spectrometry on the OpenProt resource [3]. 19 mutations (73.1%) fell exclusively in the overlapping region, when the expected value was of 10.07 ± 2.48 (38.7%), yielding a z-score of 3.60 (Fig. 1B). Additionally, we retrieved mutations within *HEY2* listed in the COSMIC catalog. 243 mutations were reported within *HEY2*, with 157 falling within the union of the canonical and the alternative ORFs. 83 mutations (52.9%) fell exclusively in the overlapping region, when the expected value was of 60.81 ± 6.10 (38.7%), yielding a z-score of 3.64 (Fig. 1B). Furthermore, looking at mutations synonymous for Q9UBP5, the COSMIC dataset contained 55 synonymous variants. Out of these, 32 variants (58.2%) clustered on the IP_145210 ORF. The expected value was 21.3 ± 3.61 (38.7%), yielding a z-score of 2.96. Hence, both datasets present a significant enrichment of genetic variants at the locus of the alternative protein IP_145210 within *HEY2*.

**Fig. 2:**
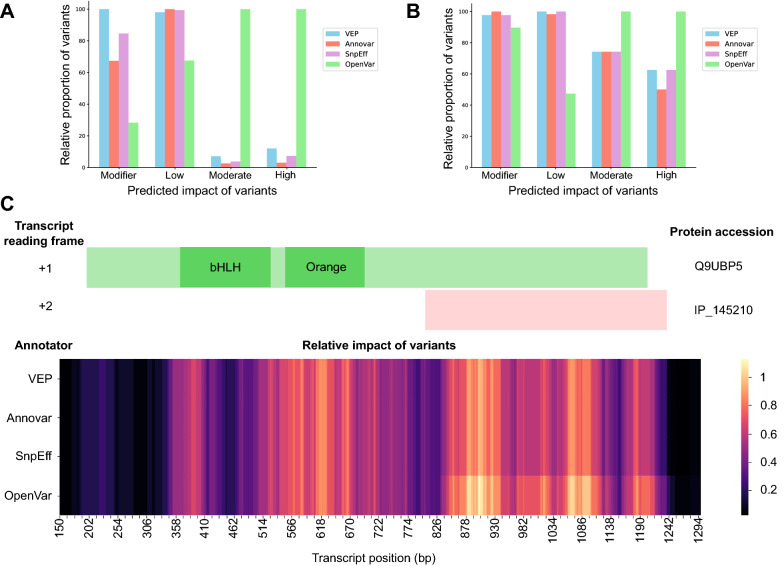
OpenVar reclassifies many low impact variants as high impact and highlights the role of non-canonical ORFs. **A** Bar chart of the relative proportion of variants in each impact category (modifier, low, moderate, high) when annotating the SynMicDB dataset with the Ensembl Variant Effect Predictor (VEP, in blue), Annovar (in red), SnpEff (in purple) or OpenVar (in green). **B** Bar chart of the relative proportion of variants in each impact category (modifier, low, moderate, high) when annotating the COSMIC catalog of variants for the *HEY2* gene with the Ensembl Variant Effect Predictor (VEP, in blue), Annovar (in red), SnpEff (in purple) or OpenVar (in green). **C** Visualization of the relative impact of genomic variants in the *HEY2* gene from the COSMIC dataset when annotated with the Ensembl Variant Effect Predictor (VEP), Annovar, SnpEff or OpenVar. The *HEY2* transcript position is indicated at the bottom (ENST00000368364.3). The position of the canonical ORF is represented in light green (UniProt: Q9UBP5; which corresponds to Ensembl: ENSP00000357348.3 or NCBI RefSeq: NP_036391.1; transcript coordinates: 198-1212) with its functional domains in dark green (bHLH: IPR011598; and Orange: IPR003650). The position of the alternative ORF is represented in light red (OpenProt: IP_145210; transcript coordinates: 805-1237)

To highlight the impact of including alternative ORFs in analyses of genomic variants, we compared the annotation of the SynMicDB and the COSMIC *HEY2* datasets when analysed with OpenVar or the most common annotators: the Ensembl Variant Effect Predictor (VEP) [14], Annovar [15] and SnpEff [11] (Fig. 2A, B). Although small differences were observed between VEP, Annovar and SnpEff, none predicted as many high impact variants than OpenVar. As the SynMicDB dataset is a database of synonymous mutations, most variants are classified as low impact with VEP, Annovar or SnpEff (Fig. 2A). By simply considering non-canonical ORFs overlapping the annotated ORF, OpenVar reclassify many low impact variants as high impact, yielding a 33.6-fold, 13.8-fold and 8.3-fold increase over Annovar, SnpEff and VEP respectively. Similarly, when considering a more heterogeneous set of variants with the COSMIC *HEY2* dataset, OpenVar offers a twofold increase over Annovar and a 1.6-fold increase over SnpEff and VEP in high impact variants (Fig. 2B). With both datasets, the increase in moderate and high impact variants observed with OpenVar comes from the reclassification of modifier and low impact variants, as visible on Fig. 2 with a relative decrease in the latter with OpenVar (Fig. 2A, B). For example, in the *HEY2* COSMIC dataset, the variant 6:g.125,759,806 T > G is located 4 nucleotides after the stop codon of the canonical ORF (Q9UBP5 with genomic coordinates 6:125,749,777–125,759,802) and is thus classified as a “modifier” impact by VEP, Annovar and SnpEff. However, it leads to a missense mutation (p.Phe137Leu) in the alternative ORF (IP_145210 with genomic coordinates 6:125,759,396–125,759,827) and is thus classified as a “moderate” impact on IP_145210 and a “modifier” impact on Q9UBP5 by OpenVar. Since the predicted impact is higher on the alternative ORF, this variant is counted as “moderate” impact on the general statistics presented on Fig. 2 (Fig. 2B).

When using OpenVar, the effect predicted on canonical ORFs is reported. For example, in the COSMIC dataset for the HEY2 gene, two variants are annotated as yielding a premature stop codon within the canonical ORF (Q9UBP5). These two variants chr6:125,759,167 C > T and chr6:125,759,503 C > T are the only two variants predicted as ‘stop_gained’ in the COSMIC catalog and with VEP, Annovar and SnpEff. With OpenVar, 4 variants are reported with a stop-gain effect: the 2 aforementioned variants for their effect on the canonical ORF, and 2 others yielding a premature stop codon in the alternative ORF (IP_145210): chr6:125,759,711 C > T and chr6:125,759,780 G > T. Table 1 highlights the gain of knowledge brought by OpenVar while retaining all information on the canonical ORF.

**Table 1:** Deep annotation of 4 genomic variants within the HEY2 gene with OpenVar

Genomic variant	Effect on the canonical ORF (Q9UBP5)				Effect on the alternative ORF (IP_145210)			
	Locus effect	Protein effect	Reported by common annotators	Reported by OpenVar	Locus effect	Protein effect	Reported by common annotators	Reported by OpenVar
chr6:125,759,167 C > T	Stop gained	R127*	Yes	Yes	5′UTR variant	–	No	Yes
chr6:125,759,503 C > T	Stop gained	R239*	Yes	Yes	5′UTR variant	–	No	Yes
chr6:125,759,711 C > T	Missense	S308L	Yes	Yes	Stop gained	Q106*	No	Yes
chr6:125,759,780 G > T	Missense	G331V	Yes	Yes	Stop gained	G129*	No	Yes

Common annotators include VEP, Annovar and SnpEff

*symbolises a stop codon

Interestingly, when looking at the relative impact of variants on HEY2 (Fig. 2C), OpenVar predicts higher impact variants towards the carboxyl end of the annotated ORF (Q9UBP5 protein), which corresponds exactly at the position of the alternative ORF (IP_145210 protein). The IP_145210 ORF in *HEY2* does not overlap with any known domain of the well-characterized Q9UBP5 protein. Q9UBP5 is a 337 amino acid long Hairy-related basic helix-loop-helix (bHLH) transcription repressor, but its functional domains span from amino acid 48 to 116 [16] (Fig. 2C). Meanwhile, the IP_145210 ORF overlaps the disordered carboxyl tail of the Q9UBP5 ORF. This observation agrees with previous reports suggesting intrinsically disordered regions are prone to host dual-coding events [7, 17, 18]. Thus, the results highlighted by OpenVar suggest these variants may be detrimental via their consequence on IP_145210 rather than the canonical Q9UBP5.

## Discussion

In our view, exploring the effect of variants on non-canonical ORFs helps understanding the complex genotype–phenotype relationships [10]. Clearly, further research is needed to functionally characterize these overlooked ORFs detected by Ribo-seq and/or mass spectrometry. Deep annotation of the ever-growing collection of sequencing datasets will help target and guide their functional characterization.

Although we believe that public genomic datasets can inform on functional ORFs currently not annotated, it does not eclipse the need for further analyses and experimentation. When interpreting the results from OpenVar, users must consider the uncertain nature of unannotated ORFs and existing biases in sequencing datasets (e.g. panels limited to currently defined coding exons).

OpenVar does not aim at prioritizing and filtering genomic variants from large sequencing data, which can be done using publicly available frameworks, such as KGGSeq [19] or FATHMM [20]. These frameworks heavily rely on prior knowledge on the genomic locus and encoded protein, which is detrimental to the identification of variants within non-annotated ORFs. Because these ORFs are not annotated, there is a critical lack of biological knowledge. However, we encourage the use of OpenVar concomitantly to such framework and emphasize the need for caution when interpreting the results from OpenVar.

We welcome user feedback and feature requests through the OpenVar GitHub repositories for the python package (https://github.com/MAB-Lab/OpenVar) and the web-based implementation (https://github.com/MAB-Lab/OpenVar_WebApp). Such feedback helps us to enhance and tailor OpenVar to the needs of the community. The OpenVar repository includes a detailed guide to installation and use, as well as a walk-through demonstration using provided example datasets. The OpenVar web-based platform repository contains easy-to-follow instructions to submit a dataset for analysis. Additionally, the repository includes detailed explanation of all output statistics, figures and files.

## Conclusions

In summary, thousands of ORFs have eluded reference annotations as recently shown by ribosome profiling and proteogenomics studies [6, 10, 21, 22]. OpenVar integrates such non-canonical ORFs in the analysis of genomic variants, unveiling new research avenues to understand the genotype–phenotype relationships.

## Methods

Here, we present a novel tool, OpenVar, to annotate and explore the impact of genomic variants using deeper genome annotations, such as OpenProt [3]. OpenVar is available as a python package and an easy-to-use web-based platform, facilitating its implementation independently of one’s computing knowledge or resources. To perform an analysis with OpenVar, one simply needs a curated variant calling file (VCF).

### Code availability

OpenVar’s codebase is open source and hosted on GitHub at https://github.com/MAB-Lab/OpenVar. The web application code is also available at https://github.com/MAB-Lab/OpenVar_WebApp.

### Data availability

OpenVar, like OpenProt, embraces the philosophy of data and results sharing to foster scientific discoveries. Thus, the OpenVar github repository (https://github.com/MAB-Lab/OpenVar) allows for download of the SynMicDB and COSMIC (H*EY2* gene) datasets that were used in this article. The SynMicDB dataset was downloaded at http://synmicdb.dkfz.de/rsynmicdb/. The COSMIC dataset was downloaded at https://cancer.sanger.ac.uk/cosmic. The following links can be used to download OpenVar results on the SynMicDB dataset when using the Ensembl annotation (SynMic_Ensembl), the NCBI RefSeq annotation (SynMic_RefSeq), the OpenProt-Ensembl annotation (SynMic_OP-Ensembl) or the OpenProt-RefSeq annotation (SynMic_OP-RefSeq). The following link can be used to download OpenVar results on the COSMIC-HEY2 dataset using the OpenProt-Ensembl annotation (COSMIC_OP).

### OpenVar output

OpenVar output contains 10 files. The output folder will contain the submitted variant calling file (VCF) as well as a text file with all analysis warning (warnings.txt). If a liftover between genome builds was required, OpenVar uses the Picard LiftoverVcf tool (GATK tools, Broad Institute) and the output folder will also contain all outputs files from the liftover. OpenVar generates a common annotated VCF file and two table files. The table named ‘annOnePerLine.tsv’) contains one line for each effect of each variant with the chromosome (‘CHROM’) and position (‘POS’) of the variant, the reference (‘REF’) and alternative (‘ALT’) allele, the description of the effect of the variant (‘ANN[*].EFFECT’), the impact category (‘ANN[*].IMPACT’), the DNA change (‘ANN[*].HGVS_C’) and protein mutation (‘ANN[*].HGVS_P’), the transcript and protein accession (‘ANN[*].FEATUREID’), alongside the gene name (‘ANN[*].GENE’). The table named ‘max_impact.tsv’ lists the maximal impact on both canonical (‘in_ref’) and non-canonical (‘in_alt’) ORFs for each variant. This table contains as many lines as variants submitted for the analysis. Each line starts with the variant name identifier (‘hg38_name’) which corresponds to the chromosome, position, reference and alternative alleles in an hg38 format. The columns ‘in_ref’ and ‘in_alt’ allow for easy filtering of variants falling only on canonical ORFs (in_ref = *true* and in_alt = *false*), only on non-canonical ORFs (in_ref = *false* and in_alt = *true*) or on both (in_ref = *true* and in_alt = *true*). The columns ‘ref_max_impact’ and ‘alt_max_impact’ list the maximal impact category on canonical and non-canonical ORFs respectively. To ease the filtering, impact categories are numbered from 0 to 3 corresponding respectively to “modifier”, “low”, “moderate” and “high”. The following columns list the transcript and proteins accessions for both the canonical and alternative ORFs alongside the DNA and protein changes. Additionally, OpenVar also outputs all the figures that can be found on the results page in a scalable vector graphics (SVG) format to ease their customization and inclusion in publications. A pickle object is also included (‘summary.pkl’). It contains all summary statistics needed to display the results on the web page in a python format. It can easily be loaded into any python script for downstream analysis with the following command: pickle.load(open(‘summary.pkl’, ‘rb’)). Please note you would need to import the pickle package first with import pickle.

### Statistical analysis of genomic variant cluster

The number of mutation expected to fall within the overlapping coding regions was calculated as a binomial distribution: $X\sim B(n, p)$ , where X is the number of mutations falling within the overlapping coding region amongst a set of *n* mutations; and *p* the probability for any mutation to fall within the overlapping region. We assume that (1) a mutation can happen equally on any nucleotide, and that (2) each mutations are independent. Under these assumptions, we can define: $p= \frac{\mathrm{length}({\mathrm{seq}}_{1} \cap {\mathrm{seq}}_{2})}{\mathrm{length}({\mathrm{seq}}_{1} \cup {\mathrm{seq}}_{2})}$ , where *seq*_*1*_ and *seq*_*2*_ are the two coding sequences. The significance of the observed value was assessed by measuring its z-score with regards to the mean and standard deviation of the predicted binomial distribution. A z-score over 2.58 is considered significant (p < 0.01).

### Relative impact of variants map

Each impact potential at a genomic position *x* is calculated as the mean of impacts from all variants in the interval x–30 bp and x + 30 bp. The maps for all annotator were normalized to the maximal impact obtained between VEP, Annovar and SnpEff.

## Data Availability

OpenVar, like OpenProt, embraces the philosophy of data and results sharing to foster scientific discoveries. Thus, the OpenVar github repository (https://github.com/MAB-Lab/OpenVar) allows for download of the SynMicDB and COSMIC (H*EY2* gene) datasets that were used in this article. The SynMicDB dataset was downloaded at http://synmicdb.dkfz.de/rsynmicdb/. The COSMIC dataset was downloaded at https://cancer.sanger.ac.uk/cosmic. The following links can be used to download OpenVar results on the SynMicDB dataset when using the Ensembl annotation (SynMic_Ensembl), the NCBI RefSeq annotation (SynMic_RefSeq), the OpenProt-Ensembl annotation (SynMic_OP-Ensembl) or the OpenProt-RefSeq annotation (SynMic_OP-RefSeq). The following link can be used to download OpenVar results on the COSMIC-HEY2 dataset using the OpenProt-Ensembl annotation (COSMIC_OP).

## Code availability

OpenVar’s codebase is open source and hosted on GitHub at https://github.com/MAB-Lab/OpenVar. The web application code is also available at https://github.com/MAB-Lab/OpenVar_WebApp.
